# Supplementary material for: The Distribution of Tryptophan-Dependent Indole-3-Acetic Acid Synthesis Pathways in Bacteria Unraveled by Large-Scale Genomic Analysis
Source: Molecules. 2019 Apr 10;24(7):1411. doi: 10.3390/molecules24071411 (PMC6479905; doi:10.3390/molecules24071411)
Supplement: Supplementary file 1 [file molecules-24-01411-s001.zip › Supplementary files/Supplementary Figure.pdf]

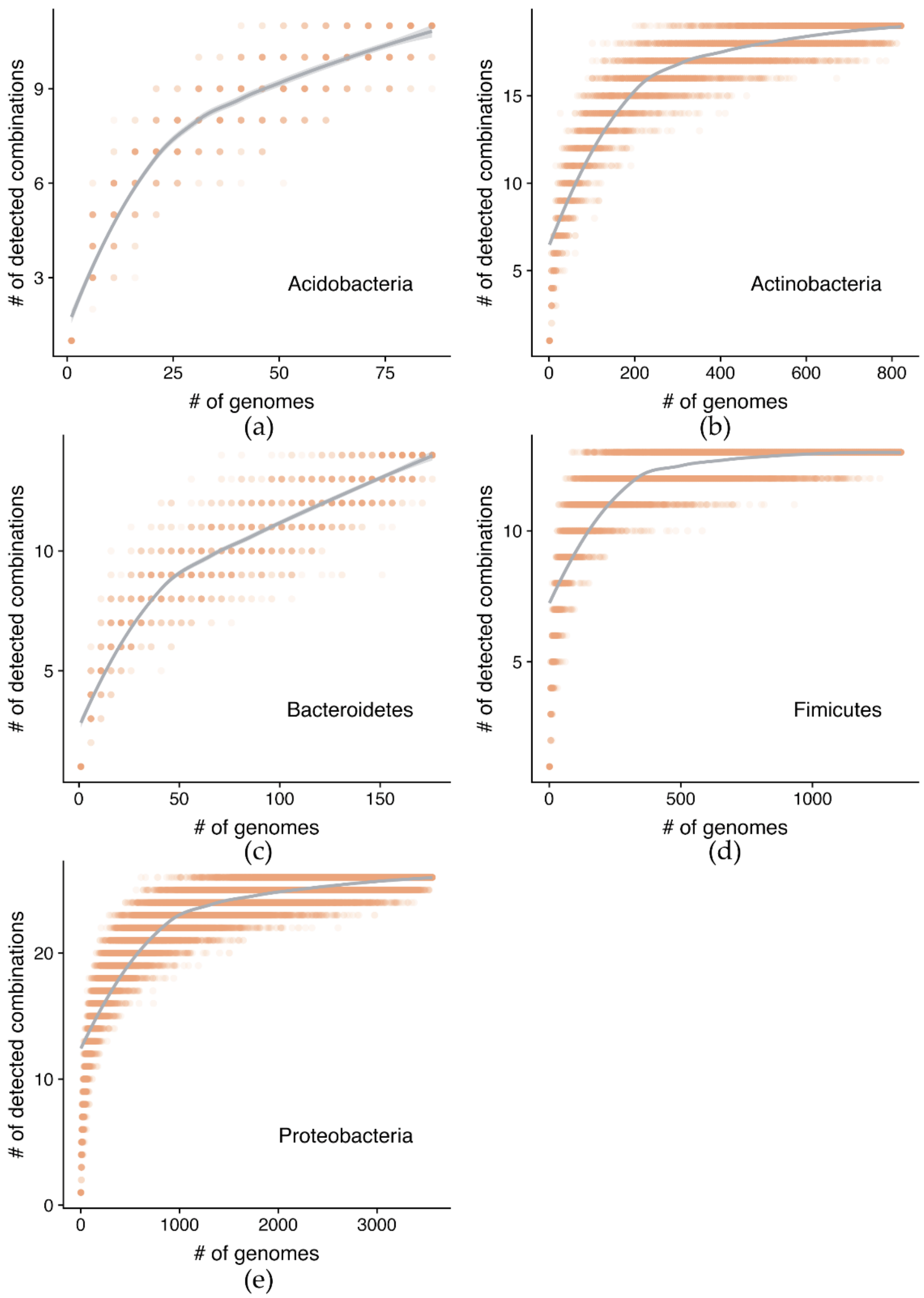

Supplementary Figure 1. Rarefaction curves displaying the number of combinations of IAA synthesis routes in each phylum. Generally, there are 6 distinct routes for IAA synthesis. But our analyses reveal that a single bacterial phylum can synthesize IAA via random combinations of 6 distinct metabolic routes (Refer Fig. 1). Each subsampling point is repeated for 50 times with a step size of 5.

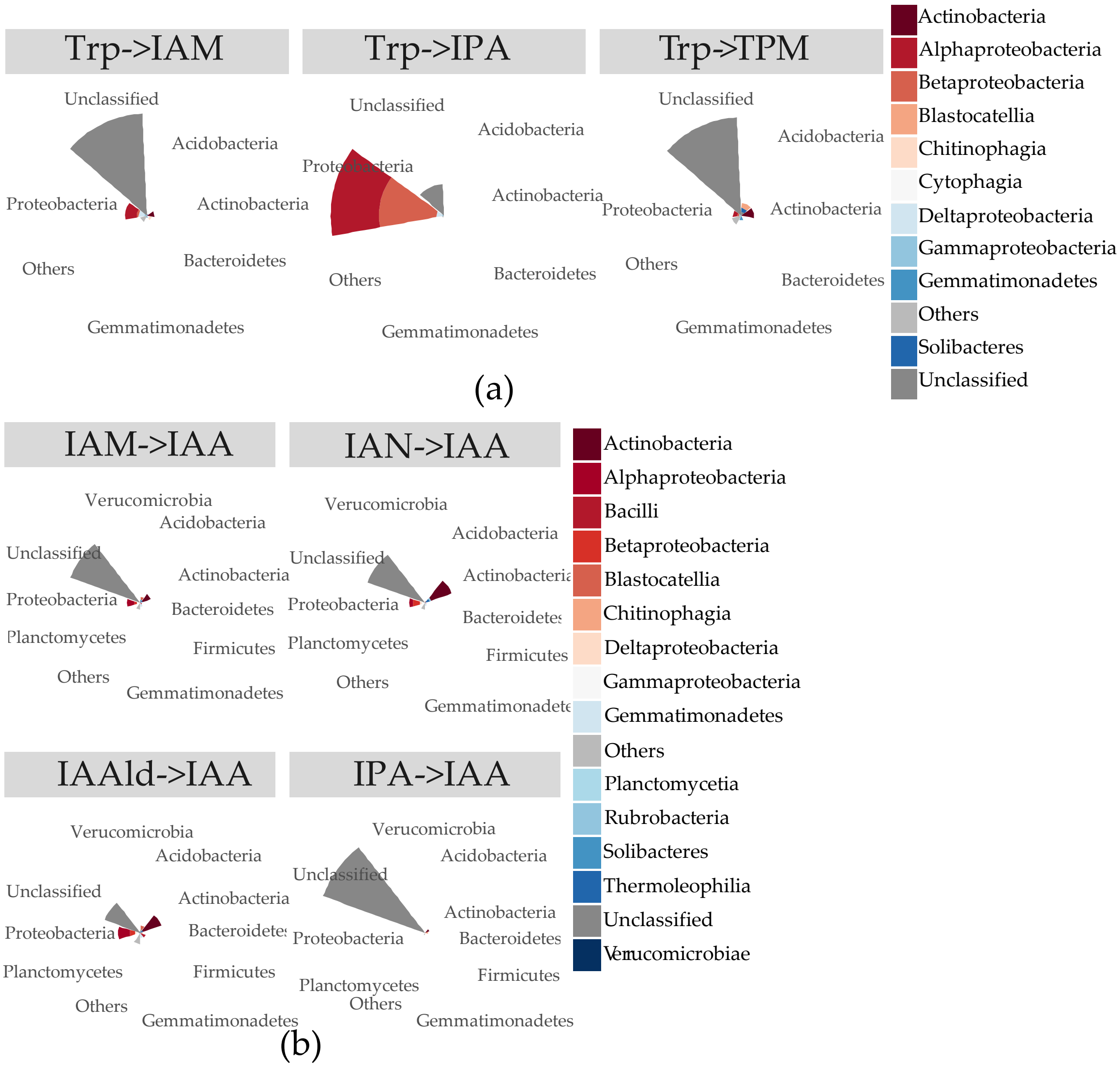

Supplementary Figure 2. The bacterial taxonomic compositions of IAA synthesis-related genes in rhizosphere. a) The taxonomic structure of Try-catalyzing enzymes in rhizosphere. b) The structure of IAA-producing enzymes in rhizosphere.

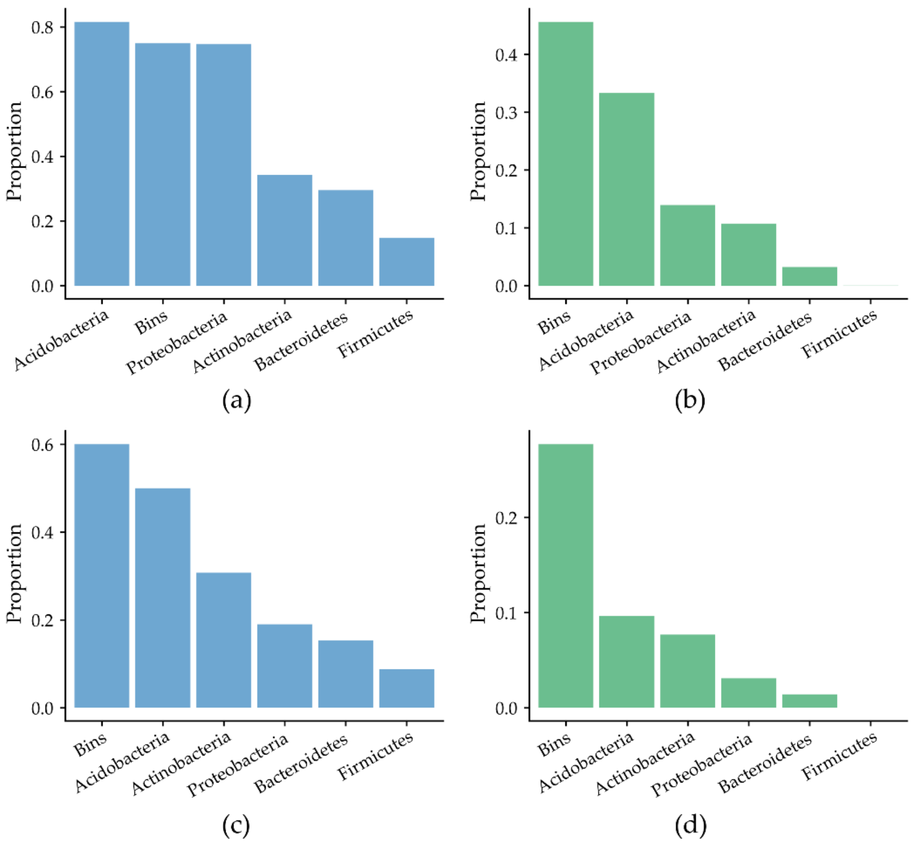

Supplementary Figure 3. The comparison of IAA synthesis capacity between metagenomic bins and retrieved genomes. a) and c) represent the proportions of genomes that possess Trp-catalyzing enzymes and complete IAA synthesis pathways in different bacteria, respectively. b) and d) represent the proportions of genomes that possess multiple Trp-catalyzing enzymes or complete IAA synthesis pathways.
